# Normalization of B Cell Subsets but Not T Follicular Helper Phenotypes in Infants With Very Early Antiretroviral Treatment

**DOI:** 10.3389/fped.2021.618191

**Published:** 2021-04-29

**Authors:** Sharon Shalekoff, Shayne Loubser, Bianca Da Costa Dias, Renate Strehlau, Stephanie Shiau, Shuang Wang, Yun He, Elaine J. Abrams, Louise Kuhn, Caroline T. Tiemessen

**Affiliations:** ^1^Centre for HIV & STIs, National Institute for Communicable Diseases and School of Pathology, Faculty of Health Sciences, University of the Witwatersrand, Johannesburg, South Africa; ^2^Empilweni Services and Research Unit, Rahima Moosa Mother and Child Hospital, Department of Paediatrics and Child Health, School of Clinical Medicine, Faculty of Health Sciences, University of the Witwatersrand, Johannesburg, South Africa; ^3^Department of Biostatistics and Epidemiology, Rutgers School of Public Health, Piscataway, NJ, United States; ^4^Department of Biostatistics, Mailman School of Public Health, Columbia University Irving Medical Center, New York City, NY, United States; ^5^ICAP at Columbia University, Mailman School of Public Health, and Department of Pediatrics, Vagelos College of Physicians and Surgeons, Columbia University Irving Medical Center, New York City, NY, United States; ^6^Gertrude H. Sergievsky Center, Vagelos College of Physicians and Surgeons, and Department of Epidemiology, Mailman School of Public Health, Columbia University Irving Medical Center, New York City, NY, United States

**Keywords:** cTfh, B cells, infants, HIV-1, early antiretroviral therapy

## Abstract

**Introduction:** Infant HIV-1-infection is associated with high morbidity and mortality if antiretroviral treatment (ART) is not initiated promptly. We characterized development of circulating T follicular helper cells (cTfh) and their relationship to naïve/memory B cell subsets in a cohort of neonates initiating ART within the first week of life.

**Methods:** Infants were diagnosed within 48 hours of birth and started ART as soon as possible. The frequency and phenotype of cTfh and B cells were analyzed at enrollment (birth −19 days) and at 4, 12, and 72 weeks of age in blood of 27 HIV-1-intrauterine-infected and 25 HIV-1 exposed uninfected (HEU) infants as part of a study in Johannesburg, South Africa. cTfh cells were divided into Tfh1, Tfh2, and Tfh17 subsets. B cell phenotypes were defined as naïve, resting memory, activated memory and tissue-like memory cells.

**Results:** HIV-1-infected infants had higher frequencies of cTfh cells than HEU infants up to 12 weeks of age and these cTfh cells were polarized toward the Tfh1 subset. Higher frequencies of Tfh1 and lower frequencies of Tfh2 and Tfh17 correlated with lower CD4+ T cell percentages. Lower frequencies of resting memory, with corresponding higher frequencies of activated memory B cells, were observed with HIV-1 infection. Importantly, dysregulations in B cell, but not cTfh cell, subsets were normalized by 72 weeks.

**Conclusion:** Very early ART initiation in HIV-1-infected infants normalizes B cell subsets but does not fully normalize perturbations in cTfh cell subsets which remain Tfh1 polarized at 72 weeks. It remains to be determined if very early ART improves vaccine antibody responses despite the cTfh and B cell perturbations observed over the time course of this study.

## Introduction

There is overwhelming consensus regarding the importance of starting antiretroviral therapy (ART) in HIV-1-infected infants as soon as they are diagnosed given the rapid disease progression and high mortality observed without treatment (1, 2). Initiation of ART at younger ages in perinatally-infected infants has also consistently been shown to reduce the size of the viral reservoir (3–6). Sporadic cases of early-treated infants who display relatively long periods of viral control after ART interruption added further hopes that early initiation of ART may protect crucial immune function (7, 8).

T follicular helper cells (Tfh) are a specialized subset of CXCR5-expressing CD4+ T cells that are crucial in the maintenance and proliferation of B cells in the germinal centers (GC) of lymphoid tissues (9). Tfh cells play a complex role in HIV-1 infection. They are highly permissive to HIV-1 infection and serve as a major reservoir for HIV (10, 11), while also being associated with broadly-neutralizing HIV antibody responses (12, 13). CXCR5+ CD4+ T cells found in peripheral blood, termed circulating Tfh (cTfh) cells (14–16), can be used as a surrogate marker for GC Tfh cells.

In healthy individuals, the majority of the B cells are naïve or resting memory B cells. However, skewing of the composition of the B cell pool occurs during HIV-1 infection. Proportions of activated memory B cells and tissue-like memory B cells are expanded in HIV-1-infected individuals, as are immature transitional B cells, while proportions of resting memory B cells are depleted [reviewed in Moir and Fauci (17)].

The immune system of infants is still developing and the composition and phenotype of cTfh cells and B cell subsets differs from that in adults, thus impacting the immune response (18, 19). Perturbations in Tfh and B cell subsets have been found in HIV-1-infected children despite ART (20–25). However, earlier ART initiation and lower viral load was associated with reduction in some of the perturbations (20, 21, 23–26). Previous studies to evaluate the impact of ART on the restoration of Tfh and B cells have not included infants starting ART very soon after birth, where the potential to improve outcomes is greater. The aim of this study was to determine if very early ART can preserve normal Tfh/B cell development. Phenotypic changes in cTfh and B cell subsets were examined over time, from birth through 72 weeks of age, among HIV-1 intrauterine-infected infants initiating ART soon after birth and were compared to a cohort of HIV-1 exposed uninfected (HEU) infants, also followed from birth, at the same clinical site in Johannesburg, South Africa.

## Materials and Methods

### Study Participants

Twenty-seven HIV-1-infected infants diagnosed within 48 h of birth and 25 HEU infants born to HIV-1-infected mothers but testing negative within 48 h of birth were enrolled and followed prospectively to at least 72 weeks of age. These infants were enrolled as part of the Latency and Early Neonatal Provision of Antiretroviral Drugs (LEOPARD) study at Rahima Moosa Mother and Child Hospital in Johannesburg, South Africa. Samples were collected between 31 August 2015 and 25 June 2018. HIV-1-infected infants were started on ART (zidovudine, lamivudine and nevirapine) as soon as possible (0–7 days) and were followed on sustained ART (27). The frequency and phenotype of cTfh and B cells were analyzed at enrollment (as close to birth as possible; all <19 days of birth) and at 4, 12, and 72 weeks of age.

### Plasma Viral Load and CD4 Counts

Plasma HIV viral loads and CD4+ T cell counts and percentages were measured at enrollment and at 72 weeks. Viral load was measured using the COBAS® AmpliPrep/COBAS® TaqMan® HIV-1 test, version 2.0 (Roche Molecular Systems, Inc., Branchburg, NJ) with a limit of detection of 20 copies/ml. CD4+ T cell counts and percentages were measured using the Trucount method (BD Biosciences, San Jose, CA).

### Flow Cytometry

EDTA-anticoagulated whole blood samples were stained within 6 h of collection. Briefly, 100 μl of whole blood was stained with CD8 PerCP (SK1), CD4 FITC (SK3), CD3 APC-H7 (SK7), CD20 APC (2H7), PD-1 BV786 (EH12.1), CCR6 BV711 (11A9), inducible costimulator (ICOS) BV650 (DX29), CD21 BV421 (B-ly4), CD45RA PE-Cy7 (HI100), CD27 PE-CF594 (M-T271) (BD Biosciences, CA), CXCR3 BV510 (G025H7) (Biolegend, San Diego, CA), and CXCR5 PE (MU5UBEE) (eBioscience, San Diego, CA) for 15 min, after which red blood cells were lysed with FACS lysing solution (BD Biosciences), washed and resuspended in FACSflow and acquired on a four laser BD LSRFortessa™ X-20 Special Order Research Product (BD Biosciences, San Jose, CA) within 4 h. CS&T beads and mid-range Rainbow Fluorescent Particles (both BD Biosciences) were run before sample acquisition. Compensation was performed for each experiment using BD™ CompBeads (BD Biosciences). Samples were analyzed using FlowJo software version 9.9.6.

### Statistical Analyses

Baseline characteristics were analyzed with descriptive statistics and compared between groups with Fisher's exact test for categorical variables and Mann-Whitney U non-parametric test for continuous variables.

For analysis for cell subsets, we conducted cross-sectional analyses with Mann-Whitney U tests to compare subsets between groups at enrollment, 4, 12, and 72 weeks. Fourteen cell subsets were analyzed at four timepoints. Multiple comparison adjustment was performed using Benjamini–Hochberg (BH) procedure controlling the false discovery rate (FDR) at level 0.05 (28). For longitudinal analysis, generalized estimating equations (GEE) were used to test for the age trend within each group.

Spearman correlation coefficients were used to describe associations between cTfh and B cell subsets and markers of HIV disease severity (CD4+ T cell percentage and viral load) at enrollment and 72 weeks and between cTfh and B cell subsets at enrollment, 4, 12, and 72 weeks. Statistical analyses were performed using R version 3.6.3 (R Core Team, 2020) and STATA version 12.1 (StataCorp., 2011).

## Results

### Characteristics of the Study Population

Characteristics of the study population are presented in Table 1. All of the HEU infants and 25 of the 27 HIV-1-infected infants received nevirapine prophylaxis. One HEU infant and one HIV-1-infected infant additionally received zidovudine prophylaxis. All infants were started on cotrimoxazole prophylaxis from 6 weeks of age. One HIV-1-infected infant died at 89 days of age and one HEU infant at 25 days of age, with a number of infants lost to follow-up. CD4+ T cell counts were available for 24 of the 27 infants at enrollment and for all 15 infants at 72 weeks. HIV viral loads were available for all of the infants at enrollment and at 72 weeks. There was a strong positive correlation between infant and maternal viral load at enrollment (*r* = 0.746, *P* < 0.001), but this was lost at 72 weeks.

**Table 1:** Characteristics of HIV-exposed uninfected (HEU) and HIV-1-infected infants.

	**HEU (*n* = 25)**	**HIV-1-infected (*n* = 27)**	***P***
Sex (male/female; n)	12/13	14/13	1
Delivery (Vaginal/cesarean; n)	23/2	19/8	0.08
Birth weight (g), median (range)	3,090 (2,360–3,780)	2,960 (1,490–4,150)	0.3
Gestational age (≥37 weeks/ <37 weeks; n)	24/1	24/3	0.6
Infant prophylaxis			
None	0	2	0.49
Nevirapine, n (%)			
Yes	25 (100%)	25 (93%)	
No	0	2 (7%)	
Zidovudine, n (%)			
Yes	1 (4%)	1 (3.7%)	
No	20 (80%)	26 (96.3%)	
Unknown	4	0	
Age at ART initiation (≤48 h/48 h −7 days; n)	NA	21/6	
Ever breastfed (yes/no, n)	19/6	23/4	0.49
Enrollment age (≤48 h/5 −19 days; n)	21/3	19/8	0.18
Baseline viral load (HIV log_10_ RNA copies/ml), median (range)	NA	4.2 (1.3-6.4)	
72 week HIV viral load (copies/ml), n (%)			
<50	NA	13 (86.7%)	
<51–1,000	NA	1 (6.7%)	
>1,000	NA	1 (6.7%)	
Baseline CD4+ T cell count (cells/μl), median (range)	ND	2,111 (1157–3904)	
72 week CD4+ T cell count (cells/μl), median (range)	ND	2,917 (1432–3695)	
Baseline % CD4+ T cells, median (range)	ND	43.58 (22.09–67.16)	
72 week % CD4+ T cells, median (range)	ND	32.65 (23.95–38.05)	
Number of infants at 4/12/72 weeks	23/22/15	24/23/15	
Maternal ART during pregnancy, n (%)	25 (100)	22 (81)	0.052
Maternal ART category, n (%)			
ART started before pregnancy and continued	6 (24%)	3 (11.1%)	
ART started during pregnancy, <12 weeks	2 (8%)	8 (29.6%)	
ART started during pregnancy, >12 weeks	17 (68%)	10 (37%)	
ART started during pregnancy, unknown time	0	1 (3.7%)	
Maternal CD4+ T cell count close to delivery (cells/μl), median (range)	479 (10–935)	366 (38–1129)	*P* = 0.167
Maternal HIV log_10_ RNA copies/ml close to delivery, median (range)	1.5 (1.3–5.66)	4.5 (1.3–5.75)	*P* = 0.0005

*NA, not applicable; ND, not done; ART, antiretroviral therapy; h, hours*.

### Differences Between HIV-1-Infected and HEU Infants Occur Mainly in the First 12 Weeks

We tested for differences in cell subsets between HIV-1-infected and HEU infants at enrollment, and 4, 12 and 72 weeks. Fourteen cell subsets (cTfh, Tfh1, Tfh2, Tfh17, quiescent Tfh, activated Tfh, naïve B cells, resting memory B cells, activated memory B cells, tissue-like memory B cells and CD4+ICOS+, CD4+PD-1+, CD8+ICOS-1+ and CD8+PD-1+ T cells) were analyzed at 4 timepoints (56 comparisons). After FDR adjustment, 16/56 comparisons were significant. Eight of these were at enrollment, three at 4 weeks, four at 12 weeks, and one at 72 weeks.

### Memory cTfh Cells Are Higher in Early Life in HIV-1-Infected Infants

**Figure 1:**
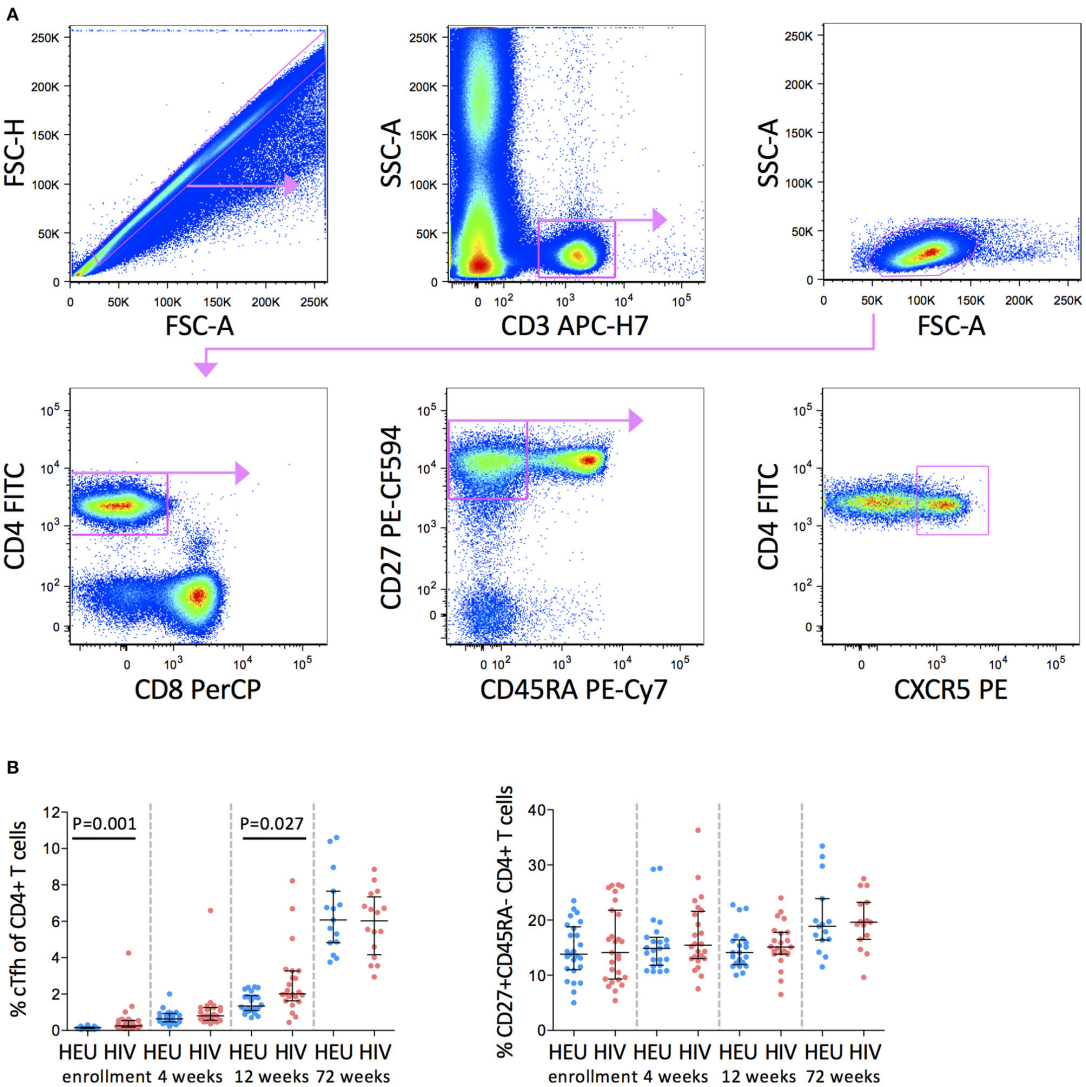
cTfh cells are increased in HIV-1-infected infants in early life. **(A)** Pseudocolor plots showing representative gating of cTfh cells. Singlets are identified using FSC-H against FSC-A followed by gating on CD3+ T cells. CD3+ T cells are further gated on low SSC-A vs low FSC-A. Subsequently, CD27+CD45RA- memory T cells are gated from CD4+ T cells and cTfh cells are identified by CXCR5 expression. **(B)** Frequencies of cTfh cells (left) and CD27+CD45RA- CD4+ T cells (right) in HEU and HIV-1-infected infants at enrollment, 4, 12, and 72 weeks. Each symbol represents an infant. Horizontal lines and error bars represent the median, 25 and 75th percentiles. The significant *P*-values are shown.

In this study, cTfh cells were defined as CD27+CD45RA-CD4+ T cells that express CXCR5 (Figure 1A). Frequencies of cTfh cells increased with increasing age in both groups. cTfh cells were, however, higher in the HIV-1-infected than HEU infants at enrollment (median 0.26 vs. 0.15%) and 12 weeks (median 2.02 vs. 1.35%) but by 72 weeks of age, these differences were no longer evident (median 6.49 vs. 6.07%) (Figure 1B).

We next investigated whether the higher frequencies of cTfh cells in HIV-1-infected infants observed here were due to higher frequencies of CD27+CD45RA-CD4+ memory T cells in the HIV-1-infected compared to the HEU infants. We found no difference in frequencies of parental CD27+CD45RA-CD4+ memory T cells between the two groups of infants suggesting that the expansions of cTfh cells up to 12 weeks in the HIV-1-infected infants was not the result of alterations in the parental population (Figure 1B).

No significant associations with CD4+ T cell percentage or HIV viral load at enrollment or 72 weeks were observed (Supplementary Figure 1).

### cTfh Cells Are Polarized Toward a Tfh1 Phenotype in HIV-1-Infected Infants

**Figure 2:**
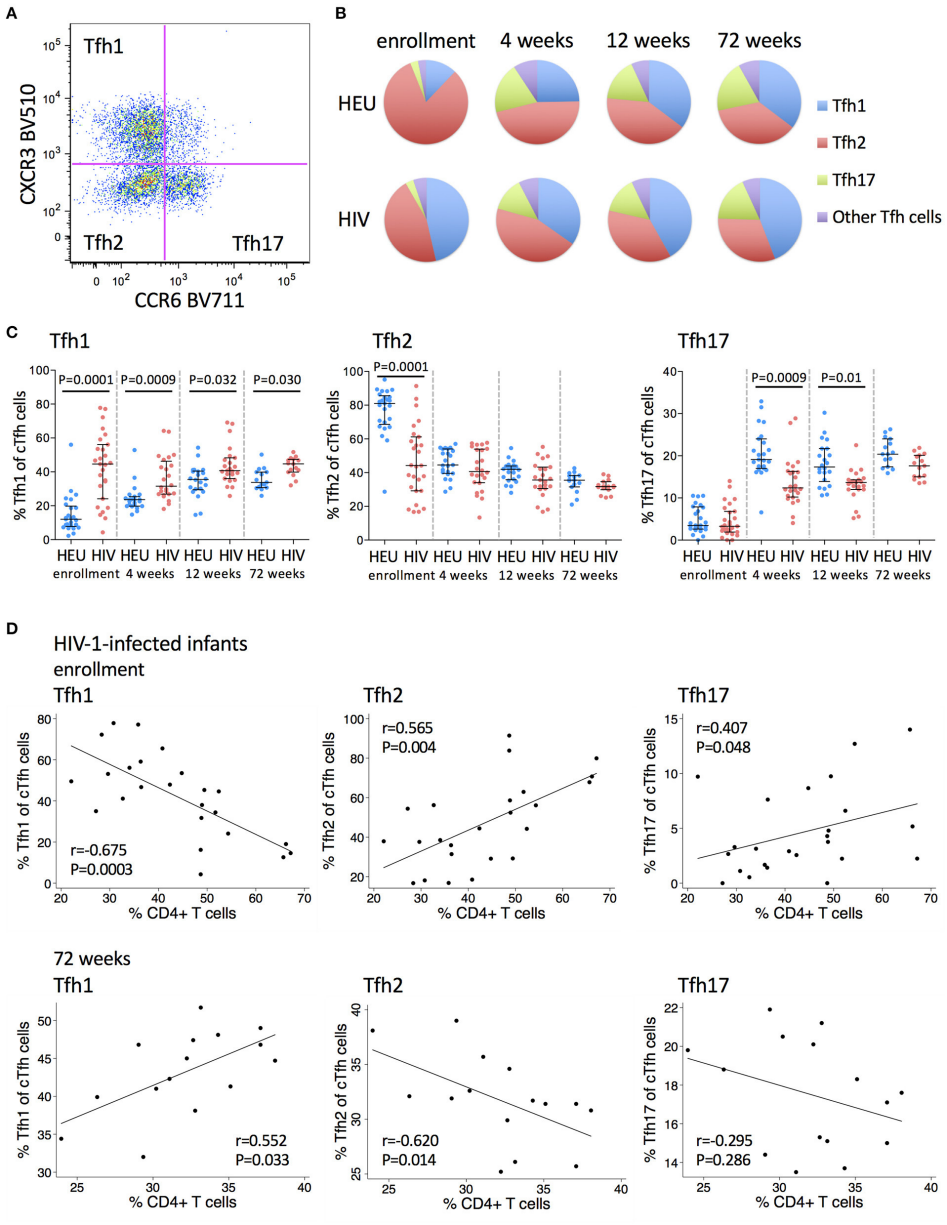
Frequencies of Tfh1 cells are increased and frequencies of Tfh2 and Tfh17 cells are decreased in HIV-1-infected infants. **(A)** A pseudocolor plot showing cTfh cells that have been subdivided into Tfh1, Tfh2 and Tfh17 subsets based on their CXCR3 and CCR6 expression. **(B)** The distribution of Tfh1 (blue), Tfh2 (red), Tfh17 (green), and the other Tfh subsets (purple) within total cTfh cells. Areas represent the medians of frequencies. **(C)** Frequencies of Tfh1, Tfh2, and Tfh17 subsets of total cTfh cells in HEU and HIV-1-infected infants at enrollment, 4, 12, and 72 weeks. Each symbol represents an infant. Horizontal lines and error bars represent the median, 25 and 75th percentiles. Significant *P*-values are shown. **(D)** Correlations between frequencies of Tfh1, Tfh2, and Tfh17 cells with % CD4+ T cells at enrollment and 72 weeks in HIV-1-infected infants. Lines are calculated using simple linear regression. Spearman rho (r) values and *P*-values are shown.

Having observed an expansion in cTfh cells in early life in HIV-1-infected infants, we next analyzed the phenotype of these cells in more detail. cTfh cells were divided into functionally distinct subsets that share features of Th1, Th2, and Th17 cells. Tfh1, Tfh2, and Tfh17 subsets were identified based on differential expression of CXCR3 and CCR6 (29) (Figure 2A).

A higher frequency of Tfh1 cells was observed in HIV-1-infected compared to HEU infants at all time points, but most markedly at enrollment (median 44.6 vs 12%) (Figures 2B,C). Age trends differed between HIV-1-infected and HEU infants. There was a steady increase in the cTfh1 subset with increasing age in HEU infants. In contrast, in the HIV-1-infected infants, there was an initial decline in the frequency of Tfh1 cells from enrollment to 4 weeks, followed by increases thereafter (Figure 2C).

A lower frequency of Tfh2 cells was observed in HIV-1-infected compared to HEU infants at enrollment (median 44.2 vs. 81%) with no differences noted at older ages (Figure 2C). Frequencies of this subset decreased with age in both groups. However, the age-decline was less pronounced in HIV-1-infected infants who started at lower levels (Figure 2C).

Frequencies of Tfh17 cells were similar in HIV-1-infected compared to HEU infants at enrollment (median 3.29 vs. 3.49%) but were lower in HIV-1-infected infants at 4 weeks (median 12.4 vs. 19.1%) and later. A striking increase in the frequency of Tfh17 cells from enrollment to 4 weeks was observed for both groups (Figure 2C).

In HIV-1-infected infants at enrollment, higher frequencies of Tfh1 cells were associated with lower CD4+ T cell percentages, whereas higher frequencies of Tfh2 cells and Tfh17 cells were associated with higher CD4+ T cell percentages. At 72 weeks, the associations were reversed with positive and negative correlations observed between Tfh1 and Tfh2 cells, respectively and CD4+ T cell percentages at this time (Figure 2D). No associations were observed between Tfh1, Tfh2 or Tfh17 cells and HIV viral load at enrollment or at 72 weeks (Supplementary Figure 2).

Collectively, these results show that cTfh cells from HIV-1-infected early treated infants remain polarized toward a Tfh1 phenotype at 72 weeks.

### Frequencies of PD-1 Expressing cTfh Cells Are Higher in HIV-1-Infected Infants

**Figure 3:**
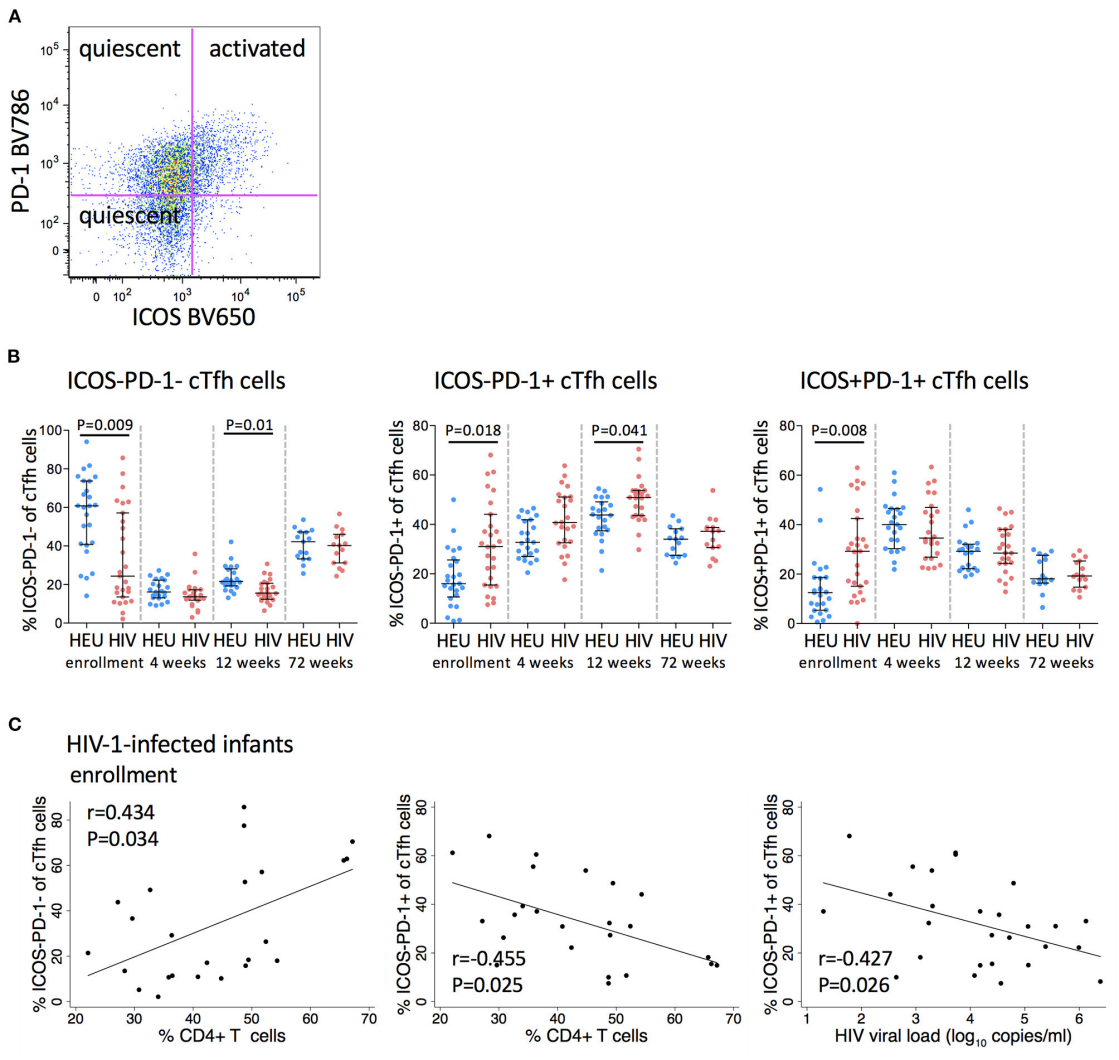
Frequencies of PD-1 expressing cTfh cells are increased in HIV-1-infected infants. **(A)** A pseudocolor plot showing cTfh cells that have been further subdivided into two quiescent subsets (ICOS-PD-1- and ICOS-PD-1+) and an activated subset (ICOS+PD-1+) based on their ICOS and PD-1 expression. **(B)** Frequencies of quiescent and activated cTfh cells in HEU and HIV-1-infected infants at enrollment, 4, 12, and 72 weeks. Each symbol represents an infant. Horizontal lines and error bars represent the median, 25 and 75th percentiles. Significant *P*-values are shown. **(C)** Correlations between frequencies of quiescent (ICOS-PD-1- and ICOS-PD-1+) cTfh cells and % CD4+ T cells and between ICOS-PD-1+ cTfh cells and HIV viral load (log_10_ copies/ml) in HIV-1-infected infants at enrollment. Lines are calculated using simple linear regression. Spearman rho (r) values and *P*-values are shown.

CXCR5+PD-1+ICOS+ Tfh cells express high levels of the proliferation marker, Ki67, suggesting that they are an activated subset of cells, whereas both PD1+ICOS- and PD1-ICOS- cTfh cells do not express Ki67, suggesting quiescence (30) (Figure 3A). ICOS-PD-1- cTfh cells declined from enrollment to 4 weeks and then increased to 72 weeks. ICOS-PD-1+ cTfh cells increased with age up to 12 weeks and declined by 72 weeks, while PD-1+ICOS+ cTfh cells increased at 4 weeks, and continued declining up to 72 weeks in both groups (Figure 3B).

Frequencies of ICOS-PD-1- cells were significantly lower (median 24.4 vs. 60.8%), whereas frequencies of both PD-1 expressing cTfh cells were significantly higher in HIV-1-infected compared to HEU infants at enrollment (ICOS-PD-1+ median 31 vs. 16%, ICOS+PD-1+ median 29.2 vs. 12.5%). This difference was maintained to 12 weeks for both subsets of quiescent cTfh cells, but not for activated cTfh cells. Importantly, by 72 weeks of age, the frequencies of both quiescent and activated cTfh cells were comparable to those observed in HEU infants (Figure 3B).

At enrollment, ICOS-PD-1- and ICOS-PD-1+ cTfh cells correlated positively and negatively, respectively with the percentage of CD4+ T cells. Surprisingly, ICOS-PD-1+ cTfh cells also correlated *negatively* with viral load (Figure 3C).

### Frequencies of Resting Memory B Cells Are Lower and Activated Memory B Cells Are Higher in HIV-1-Infected Infants

**Figure 4:**
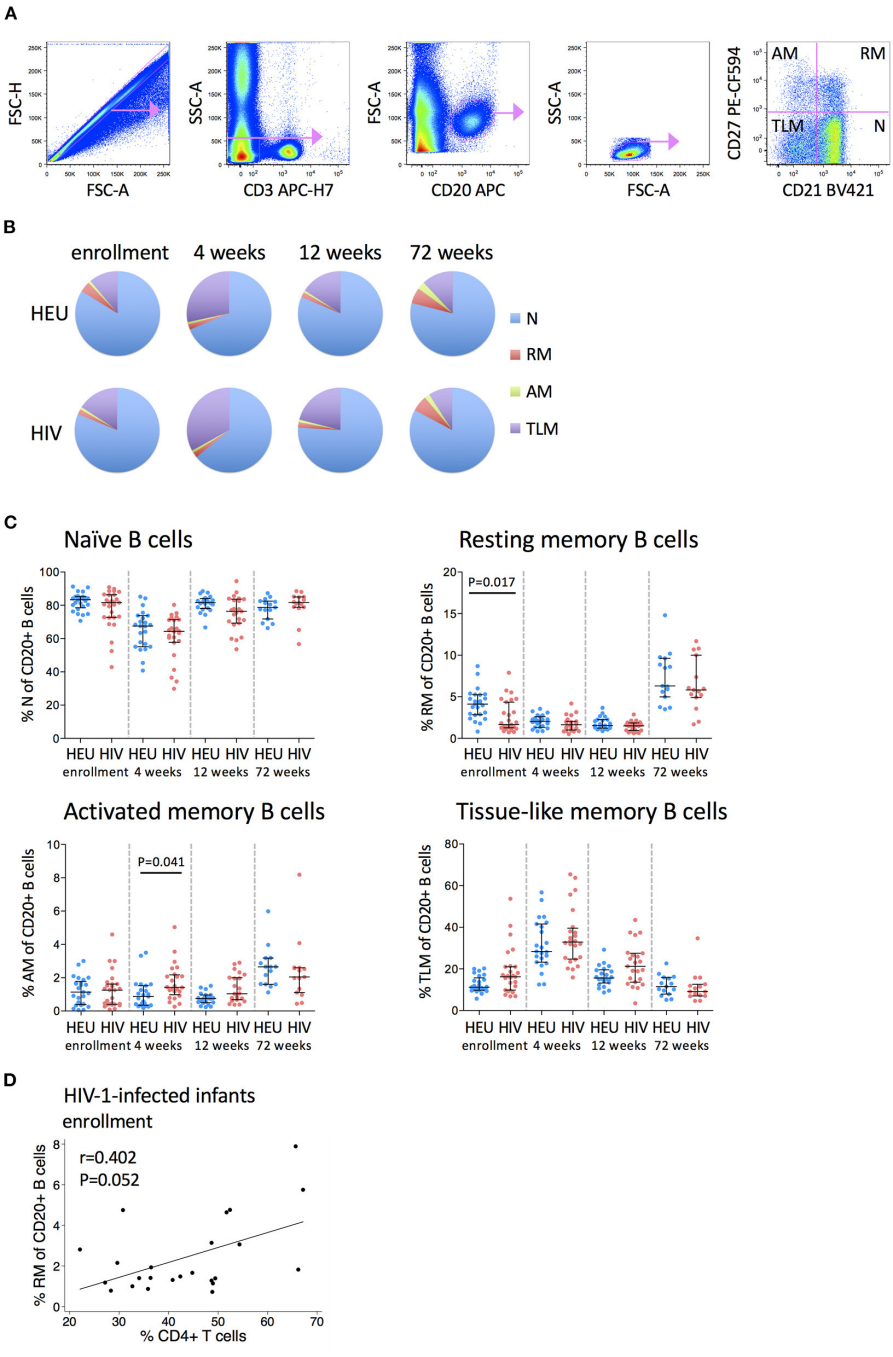
Frequencies of B cell subsets are altered in HIV-1-infected infants. **(A)** Pseudocolor plots showing representative gating of B cell subsets. Singlets are identified using FSC-H against FSC-A. B cells are identified by CD20 gating of CD3- cells, followed by gating on low SSC-A vs. low FSC-A. B cell subsets are subdivided into naïve (N), resting memory (RM), activated memory (AM), and tissue-like memory (TLM) B cells based on their CD27 and CD21 expression. **(B)** The distribution of N (blue), RM (red), AM (green), and TLM (purple) within total CD20+ B cells in HEU and HIV-1-infected infants at enrollment, 4, 12, and 72 weeks. Areas represent the medians of percentages. **(C)** Frequencies of naive, resting memory, activated memory and tissue-like memory B cells in HEU and HIV-1-infected infants at enrollment, 4, 12, and 72 weeks. Each symbol represents an infant. Horizontal lines and error bars represent the median, 25 and 75th percentiles. Significant *P*-values are shown. **(D)** Correlation between frequencies of resting memory (RM) B cells and % CD4+ T cells at enrollment in HIV-1-infected infants. Lines are calculated using simple linear regression. Spearman rho (r) values and *P*-values are shown.

B cells can be divided into naïve, resting memory, activated memory and tissue-like memory subsets based on their differential expression of CD21 and CD27 (Figure 4A). Naïve B cells constitute the largest B cell subset (~80%), followed by tissue-like memory B cells, resting memory and then activated memory B cells (~1–3%) (Figure 4B).

Each B cell subset displayed a distinctive age-related trajectory that was consistent regardless of infant HIV status. Naïve B cells declined from enrollment to 4 weeks and then returned to baseline levels at 12 and 72 weeks. Resting memory B cells declined from enrollment to 12 weeks and increased at 72 weeks. At enrollment, HIV-1-infected infants had lower frequencies of resting memory B cells than HEU infants (median 1.66 vs. 4.12%) but by 4 weeks of age, these differences were no longer apparent. Activated memory B cells were reasonably stable through 12 weeks and increased at 72 weeks with higher levels in HIV-1-infected infants at 4 weeks (median 1.43 vs. 0.87%). Tissue-like memory B cells increased from enrollment to 4 weeks and then returned to baseline levels at 12 and 72 weeks. All B cell subsets were normalized by 72 weeks of age (Figure 4C).

Higher frequencies of resting memory B cells were associated with a trend toward higher CD4+ T cell percentage in HIV-1-infected infants at enrollment (Figure 4D). No associations were observed between B cell subsets and HIV viral load at enrollment or at 72 weeks.

### HIV-1 Infection Alters Relationships Between cTfh and B Cell Subsets

**Figure 5:**
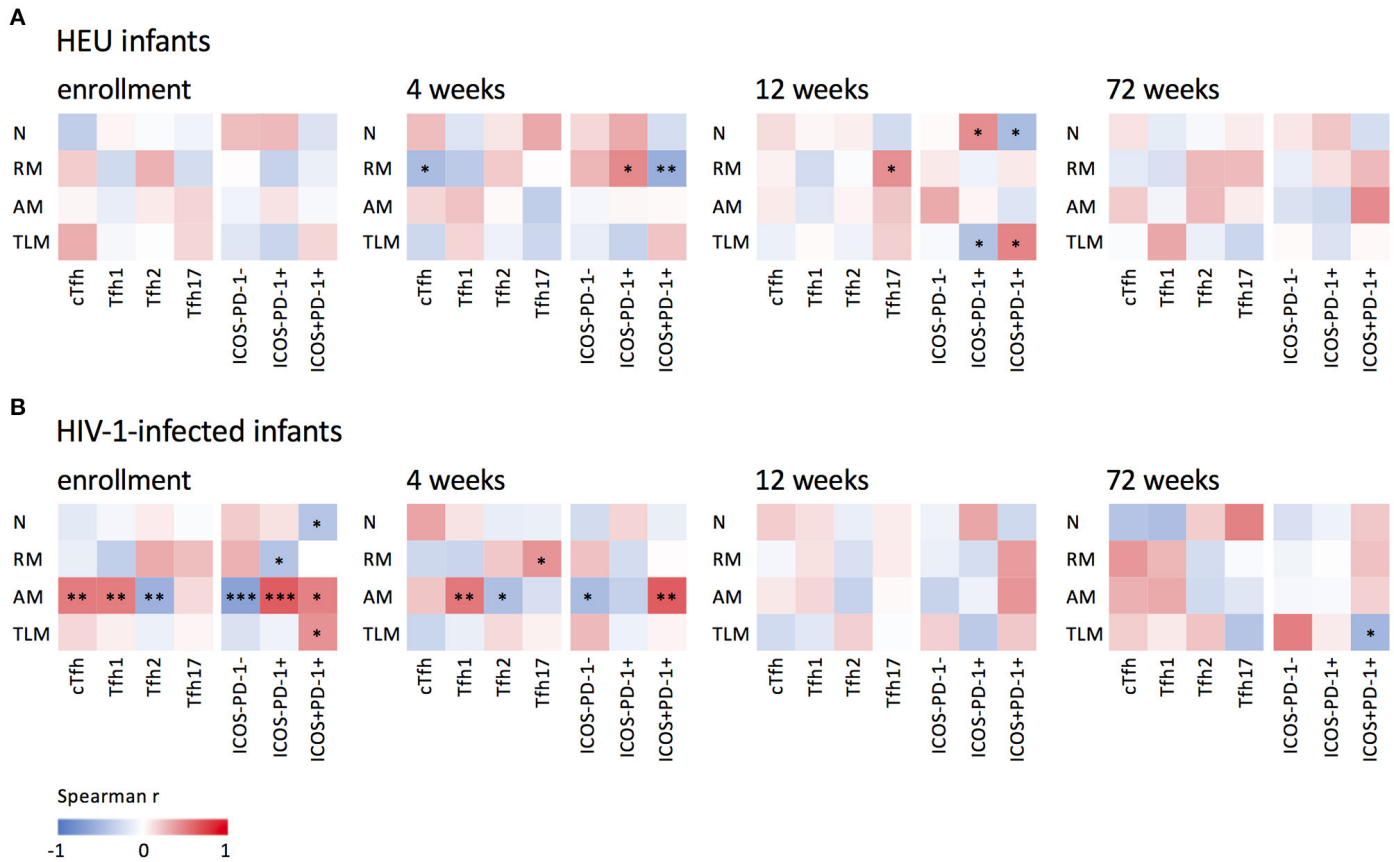
Associations between B and cTfh cells. Correlations between naïve (N), resting memory (RM), activated memory (AM), and tissue-like (TLM) memory B cells and cTfh, Tfh1, Tfh2 and Tfh17 subsets and quiescent (ICOS-PD-1- and ICOS-PD-1+) and activated (ICOS+PD-1+) Tfh cells in **(A)** HEU infants and **(B)** HIV-1-infected and at enrollment, 4, 12, and 72 weeks. ***P < 0.001, **P < 0.01, *P < 0.05.

Tfh cells are critical in B cell differentiation (9) and the frequencies of cTfh cells have been associated with the quality of B cell responses (12, 13, 21, 31). Figure 5 shows associations between cTfh, Tfh1, Tfh2, Tfh17, quiescent and activated cTfh cells and B cell subsets. Interestingly, in the HEU infants (Figure 5A), associations between frequencies of Tfh and B cell subsets occurred at 4 and 12 weeks of age, whereas in the HIV-1-infected infants (Figure 5B) they occurred almost exclusively at birth and 4 weeks. Additionally, in the HEU infants, significant associations were found between Tfh cells and all B cell subsets with the exception of the activated memory B cell subset. However, in the HIV-1-infected infants, two thirds of the associations were found with activated memory B cells. cTfh (enrollment: *r* = 0.503, *P* = 0.008), Tfh1 (enrollment: *r* = 0.501, *P* = 0.008 and 4 weeks: *r* = 0.519, *P* = 0.009), ICOS-PD-1+ (enrollment: *r* = 0.624, *P* = 0.0005) and ICOS+PD-1+ (enrollment: *r* = 0.483, *P* = 0.011 and 4 weeks: *r* = 0.620, *P* = 0.001) correlated positively and Tfh2 (enrollment: *r* = −0.548, *P* = 0.003 and 4 weeks: *r* = −0.450, *P* = 0.0273) and ICOS-PD-1- (enrollment: *r* = −0.657, *P* = 0.0002 and 4 weeks: *r* = −0.475, *P* = 0.019) correlated negatively with activated memory B cells. Only one significant association was observed at 72 weeks. This may be as a result of normalization of the frequencies of Tfh and B cell subsets and/or reduced sample numbers at 72 weeks.

## Discussion

This study was undertaken to gain insights into whether very early initiation of ART in intrauterine HIV-1-infected infants would prevent the HIV-1 related perturbations in cTfh and B cell subsets that have been observed in HIV-1-infected children despite treatment (20, 21, 23, 25, 26). We found HIV-associated perturbations in both cTfh and B cell subsets even among these very early treated children. An increased frequency of cTfh cells, with skewing toward a predominance of Tfh1 cells, over Tfh2 and Tfh17 cells, was observed. Additionally, we observed decreased frequencies of resting memory and increased frequencies of activated memory B cells. Of significance is that, by 72 weeks, the frequencies of the B cell subsets in the HIV-1-infected infants were comparable to those in the HEU infants, whereas alterations in cTfh cell subsets were still observed at the end of the study period.

In this study, skewing of cTfh and B cell subsets was already evident at enrollment. Although the timing of intrauterine infection cannot be established and is likely to be quite late in pregnancy (32), our data show that this early infection is sufficient to lead to perturbations in cTfh and B cell subsets that could be detected at enrollment. With early ART, by 72 weeks of age, only the Tfh1 cell subset had not normalized compared to that observed in HEU infants. Almost all the early-treated infants (93%) had HIV viral loads <100 copies/ml at 72 weeks, supporting the inference that the observed normalization of the frequencies of the B cell subsets is as a result of very early and sustained ART.

As far as we are aware, this is the first longitudinal study evaluating age-related changes in cTfh cell subsets in early life in any population. There are also, to our knowledge, no studies analyzing cTfh cell subsets in HIV-1-infected infants shortly after birth, prior to ART initiation, with longitudinal follow-up. Other studies have compared children with chronic infection by high or low viraemia (20, 21, 25), or children who are on ART to those not on ART (23). Here, we found that the frequency of cTfh cells is increased in HIV-1-infected compared to HEU children, contrasting with previous reports (20, 21, 23, 25). However, these previous studies were performed in older children, with wide age ranges (1–17 years) and with later ART initiation. Importantly, children who started ART earlier were found to have higher cTfh cells than those who started later and these cTfh frequencies increased with ART duration (23). Upon greater scrutiny of cell subsets which constitute cTfh cells, we observed higher frequencies of Tfh1 cells and lower frequencies of Tfh2 and Tfh17 cells in HIV-1-infected compared to HEU infants before the initiation of ART. Following sustained ART through to 72 weeks of age, there was normalization in the frequencies of these cell subsets with the exception of Tfh1 cells which remained significantly increased compared to HEU infants. We are unaware of studies analyzing Tfh1, Tfh2, and Tfh17 subsets in infants, but our findings are in agreement with a study of acute HIV-1 infection in adults which found increased frequencies of Tfh1 and Tfh2 cells and decreased frequencies of Tfh1-17 and Tfh17 cells compared to uninfected adults (33). Our findings are also consistent with a study of chronic HIV-1 infection in adults which found Tfh1 skewing of Gag-specific cTfh cells which was maintained for at least 3 years after ART initiation (34). We further found that, in early life, elevated Tfh1 cell subsets were associated with lower percentages of CD4+ T cells and with skewing of B cells toward more activated and terminally differentiated subsets. Strikingly, Tfh1, Tfh2, and Tfh17 cells each showed opposite relationships with percentage of CD4+ T cells at enrollment compared to 72 weeks. A more normalized response by 72 weeks was characterized by reduced inter-individual variability for both Tfh1 and Tfh2 subsets compared to enrollment, a median frequency of Tfh1 subsets which did not differ between enrollment and 72 weeks, while the frequency of Tfh2 subsets declined, and an age dependent decrease in the percentage of CD4+ T cells from enrollment to 72 weeks. We were unable to ascertain if these same relationships are also evident among HEU infants as CD4+ T cell percentage data was not available. However, by 72 weeks CD4+ T cell percentages of the HIV-1-infected infants were similar to reference ranges available for healthy uninfected children (35–37), suggesting that these relationships would also likely be maintained in the HEU infants.

The role of CXCR3+ Tfh1 and CXCR3- Tfh2 and Tfh17 subsets with respect to humoral immunity is not clear cut. *In vitro* studies have shown that CXCR3- cTfh cells were efficient in inducing naïve B cells to undergo isotype switching and in promoting immunoglobulin secretion (12, 29, 38), whereas CXCR3+ cTfh cells were deficient in providing help to naïve B cells but were able to help memory B cells (39). In HIV-1 progressors, circulating CXCR5+CXCR3-PD-1+ Tfh cells correlated with development of HIV-1-specific neutralizing antibodies (12) and similarly, in HIV-1-infected children (IQR 6.6–8.9 years), CXCR5+CXCR3-PD-1+ cTfh cells correlated with the breadth of neutralizing antibodies (40). However, in HIV-1 controllers, CXCR5+CXCR3+PD-1^low^ CD4+ T cells were associated with increased HIV-1 neutralizing antibody breadth (41) and in acute HIV infection, frequencies of CXCR3+ Tfh1 cells correlated positively with p24 plasma IgG titers at 1 year post infection (33). Moreover, the frequencies of CXCR3+ cTfh cells positively correlated with neutralizing antibody responses in HCV-infected individuals (42). Therefore, the role of CXCR3- and CXCR3+ cTfh subsets in the development of protective antibody responses may be situation dependent. Thus, the impact of Tfh1 polarization observed in the very young infants in our study, on the development of HIV-1-specific neutralizing antibodies, is unknown and requires further investigation.

We found a decreased frequency of ICOS-PD-1- cTfh cells with a concomitant increased frequency of ICOS-PD-1+ and ICOS+PD-1+ expressing cTfh cells in early life in HIV-1-infected compared to HEU infants. This is consistent with one study (23) and not with another (21). ICOS+PD-1+ cTfh cells express the lowest levels of CCR7, ICOS-PD-1+ express intermediate levels and ICOS-PD-1- cTfh cells express the highest levels (43). As an increase in CXCR5 together with decrease in CCR7 is needed for cTfh cells to migrate into B cell follicles (44), differing levels of CCR7 expression may reflect differing capacities of these cell subsets to traffic to B cell follicles. It is, therefore, possible that in these infants, in early life, there is an altered capacity of cTfh cells to traffic to B cell follicles. Similarly to findings from previous studies in adults (45) and children (23), we found increased PD-1 expression in HIV-1-infected infants correlated with lower percentages of CD4+ T cells. This may possibly be due to PD-1-induced exhaustion and apoptosis of CD4+ T cells. However, markers such as CD57 to define senescence were not included in this study. Additionally, it has been shown that PD-1+ cTfh cells are highly permissive to HIV-1 and that they contribute to HIV reservoirs in individuals treated with ART (11). The fact that PD-1+ cTfh cells correlated inversely with both the percentage of CD4+ T cells and with viral load is unexpected. We hypothesize that this paradoxical finding may be due to effects of maternal ART. In the same cohort overall, we observed a strong relationship between maternal viral load at delivery and infant viral load pre-ART (46). Thus, the impact of maternal ART on infant viral load may make infant viral load a weak marker of disease progression. Since pre-ART infant CD4+ T cell percentage is not similarly affected, this is a more informative marker of disease severity regardless of maternal ART.

Our finding of decreased frequencies of resting memory and increased frequencies of activated memory B cells in HIV-1-infected compared to HEU infants confirms results from other studies (21, 23, 24). Importantly, ART initiation, rapidly restored resting memory cells to normal levels by 4 weeks of age despite lower levels at enrollment in HIV-1-infected infants. Interestingly, the expansion of frequencies of cTfh, Tfh1, ICOS-PD-1+, and ICOS+PD-1+ cells at enrollment and Tfh1 and ICOS+PD-1+ cells at 4 weeks correlated positively with activated memory B cells suggesting a role for cTfh cells in the skewing of B cell differentiation toward a more activated phenotype. An important strength of our study compared to other studies in children, with the exception of one (26), is that our study is longitudinal. Furthermore, the precise age-matching and narrow age range of participants in our study prevents confounding by age. Thus, very early ART, in this study, appears to have minimized HIV-related B cell perturbations and adds to the growing body of evidence on the impact of ART, at the earliest stages of infection, in children (26, 47) and in adults (29), on the preservation of the integrity of the humoral immune response.

It is unlikely that the early dysregulations of cTfh and B cells as observed in this study would have negative implications for vaccine responses in early life, as studies of later administration of ART do not support this. A South African study evaluated the immunogenicity of the combined diphtheria, tetanus, pertussis and the monovalent HBV vaccines given at 6, 10, and 14 weeks of age in HIV-1-infected infants initiated on ART 4 days prior to the first vaccination and in a deferred ART group (48). Although HIV-1-infected children had lower geometric mean concentrations of vaccine-specific antibodies, the proportion of HIV-1-infected children, whether in the early or deferred treatment group, had seroprotective responses similar to those in uninfected infants at 1 month after the third dose of vaccine. Additionally, an Italian study found that children initiating ART within the first year of life developed and maintained protective antibody levels to measles and tetanus vaccination (47). Together these results suggest the preservation of the vaccine humoral responses as a result of early ART initiation. Whether very early ART might improve some vaccines-specific responses remains to be established.

The limitations of this study include the fact that we did not include HIV-1 unexposed uninfected (HUU) infants due to the difficulty of justifying blood draws in HUU infants. We cannot assume that maternal HIV-1-infection has no influence on cTfh and B cells in HEU infants, and comparison with HUU infants would inform this question. However, HEU infants remain an informative control group for HIV-1-infected infants by virtue of being born to HIV-1-infected mothers—thus many factors are common to both groups e.g., passive transfer of maternal HIV antibodies and maternal ART exposure. Phenotypic differences have been found in T cell subsets between HEU and HUU children due to exposure to HIV-1 and/or to ART (49). However, studies comparing the frequencies of B cell subsets, either found no difference in the frequencies any of the subsets studied (50) or reduced frequencies of only resting memory B cells in HEU compared to HUU children (51). Another limitation is that we did not adjust *P*-values for multiple comparisons when correlations of cTfh and B cell subsets with markers of disease progression were analyzed, or when we looked for associations between cTfh and B cell subsets. The longitudinal follow-up of infants, at four different timepoints, resulted in a large number of comparisons. Therefore, multiple comparison adjustment could potentially result in important associations being overlooked. Other limitations include some variation in enrollment age, the smaller sample size at 72 weeks due to loss to follow up, and the long interval between the sampling at 12 and 72 weeks. There are also limitations in the use of cTfh cells as surrogate markers of GC Tfh cells. Although CXCR5 is constitutively expressed on cTfh, CXCR5 expression is not limited to cTfh cells and is transiently expressed by recently activated CD4+ T cells (52). Further, cTfh cells differ from bona fide GC Tfh cells in that they express low levels of transcription factor B cell lymphoma 6 (Bcl-6) (39, 53), the Tfh lineage-defining transcription factor (54–56). Moreover, analyses performed on peripheral blood may not accurately reflect what is happening in the germinal centers and lymphoid tissue.

Our findings add to the current body of knowledge of cTfh and B cell subsets in children living with HIV. Very early ART initiation, has allowed for almost normal development of B cell subsets and as well as improvement in the perturbations of cTfh cell subsets. These findings support recent guidelines for routine birth testing and immediate ART initiation in HIV-1-infected infants. Whether the altered polarization of the cTfh cell subsets is restored to that observed in HEU infants, with a longer duration of ART, remains to be established. To what extent the polarization of the cTfh cells toward the Tfh1 cell subset impacts on the immune response to HIV-1 as well as persistence of the HIV reservoir is unknown and merits further investigation.

## Data Availability Statement

The raw data supporting the conclusions of this article will be made available by the authors, without undue reservation.

## Ethics Statement

The studies involving human participants were reviewed and approved by Human Research Ethics Committee (HREC) of the University of the Witwatersrand and the Institutional Review Board (IRB) of Columbia University. Written informed consent to participate in this study was provided by the participants' legal guardian/next of kin.

## Author Contributions

ShS developed the immunophenotyping flow cytometry panels, analyzed, and interpreted the data. ShS, BD and SL conducted the immunophenotyping assays. RS was involved in clinical management and interpretation of data. StS contributed to data management and interpretation of data. ShS, SW, and YH performed the statistical analyses. EA contributed to study design and interpretation of data. LK designed the study, obtained funding, was involved in management and oversight, analysis and interpretation of data. CT contributed to study design and funding, laboratory supervision, analysis, and interpretation of data. ShS, LK, and CT wrote the manuscript, which was reviewed and edited by all authors. All authors contributed to the article and approved the submitted version.

## Conflict of Interest

The authors declare that the research was conducted in the absence of any commercial or financial relationships that could be construed as a potential conflict of interest.
