# Supplementary material for: Normalization of B Cell Subsets but Not T Follicular Helper Phenotypes in Infants With Very Early Antiretroviral Treatment
Source: Front Pediatr. 2021 Apr 29;9:618191. doi: 10.3389/fped.2021.618191 (PMC8118125; doi:10.3389/fped.2021.618191)
Supplement: Supplementary file 1 [file Data_Sheet_1.PDF]

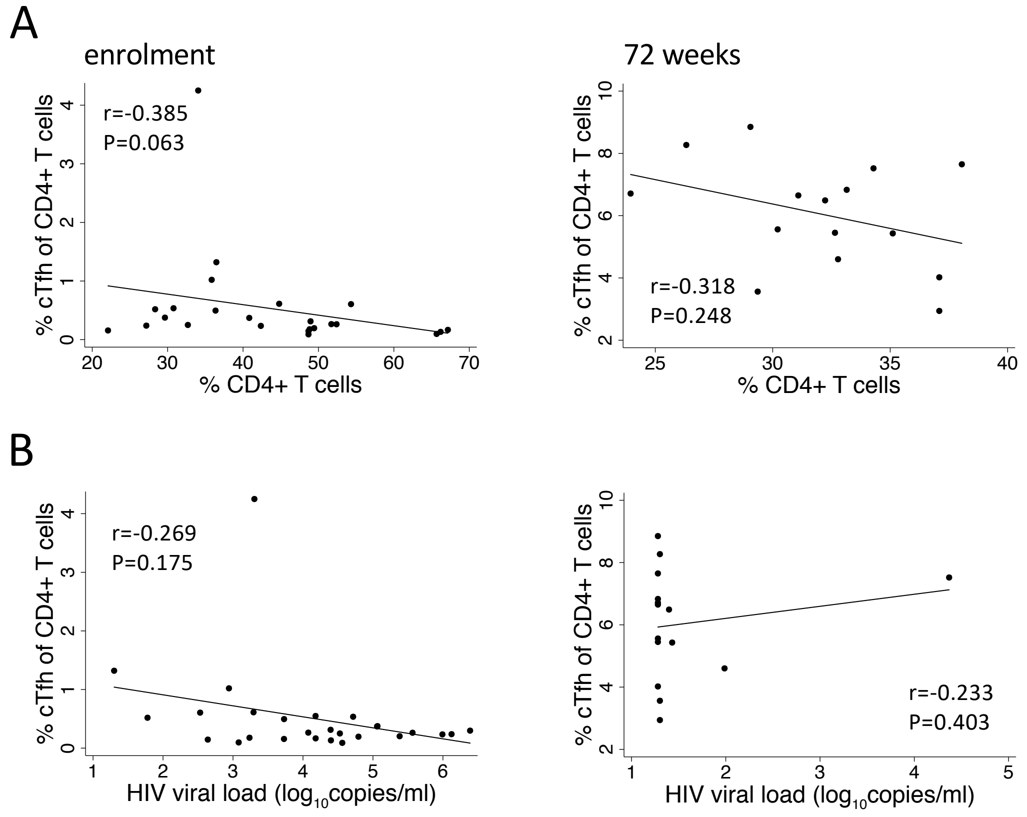

SUPPLEMENTARY FIGURE 1. Correlations between frequencies of cTfh cells and % CD4+ T cells **(A)** and HIV viral load ($\log_{10}$ copies/ml) **(B)** at enrollment and 72 weeks in HIV-1-infected infants. Lines are calculated using simple linear regression. Spearman rho (r) values and P values are shown.

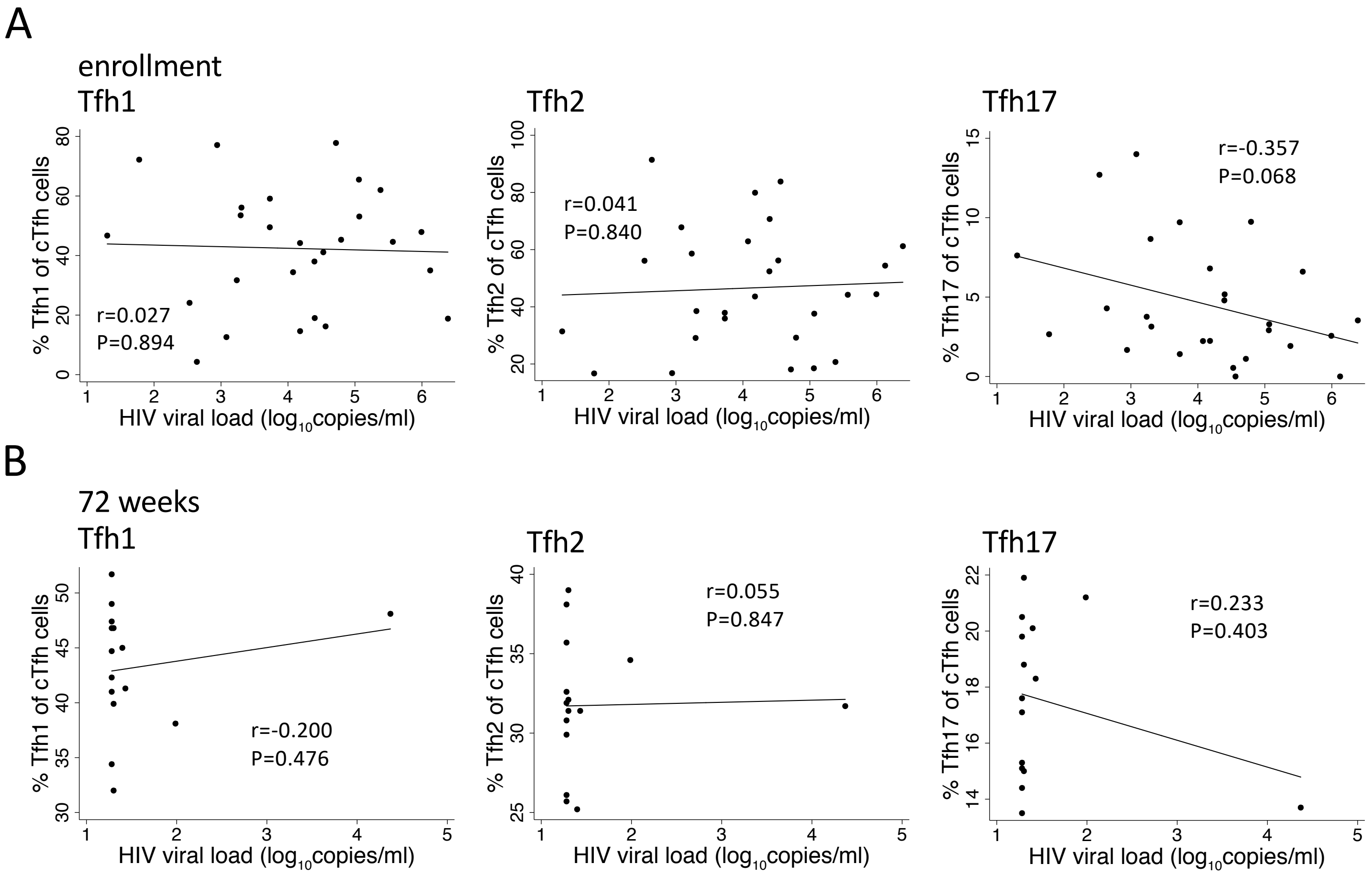

SUPPLEMENTARY FIGURE 2. Correlations between frequencies of Tfh1, Tfh2 and Tfh17 and HIV viral load (log_{10} copies/ml) at enrollment **(A)** and 72 weeks **(B)** in HIV-1-infected infants. Lines are calculated using simple linear regression. Spearman rho (r) values and P values are shown.
